# Protocol for Establishing a Multiplex Image-based Autophagy RNAi Screen in Cell Cultures

**DOI:** 10.21769/BioProtoc.2540

**Published:** 2017-09-05

**Authors:** Jennifer Jung, Christian Behrends

**Affiliations:** 1 Institute of Biochemistry II, Goethe University School of Medicine, Frankfurt, Germany; 2 Munich Cluster for Systems Neurology (SyNergy), Ludwig-Maximilians-University Munich, Munich, Germany

**Keywords:** siRNA screen, Immunostaining, Immunofluorescence, Autophagy

## Abstract

Autophagy is a recycling pathway, in which intracellular cargoes including protein aggregates and bacteria are engulfed by autophagosomes and subsequently degraded after fusion with lysosomes. Dysregulation of this process is involved in several human diseases such as cancer or neurodegeneration. Hence, advancing our understanding of how autophagy is regulated provides an opportunity to explore druggable targets and subsequently develop treatment strategies for these diseases. To identify novel autophagy regulators, we developed an image-based phenotypic RNAi screening approach using autophagic marker proteins at endogenous levels (Jung *et al*., 2017). In contrast to previously performed autophagy screens, this approach does not use overexpressed, tagged autophagy marker proteins but rather detects autophagic structures at endogenous levels. Furthermore, we monitored early and late phases of autophagy in parallel while other screens employed only a single autophagosome marker mostly GFP-LC3B. Here, we describe this multiplex screening protocol in detail and discuss general considerations about how to establish image-based siRNA screens.

## Background

Autophagy is an intracellular quality and quantity control pathway by which diverse cytosolic material such as pathogens, organelles or parts thereof, proteins and other macromolecules are engulfed by double membrane structures coined autophagosomes and delivered for bulk lysosomal degradation upon fusion of autophagosomes with lysosomes. Formation of autophagosomes and their maturation to autolysosomes is a highly regulated process. Among the AuTophaGy-related (ATG) genes initially identified in yeast is the ubiquitin-like protein Atg8, which exerts its function in a highly localized manner through its reversible conjugation to the phospholipid phosphatidylethanolamine (PE) located in the recipient autophagosome. Human cells contain six ATG8 family members that can be grouped into two subfamilies: i) microtubule-associated proteins 1A/1B light chain 3A (LC3A), LC3B and LC3C and ii) γ-aminobutyric acid receptor-associated protein (GABARAP), GABARAPL1 and GABARAPL2 (Slobodkin and Elazar, 2013). Given the fact that yeast only harbors one Atg8 isoform, it is unclear whether LC3 and GABARAP proteins are functionally redundant or have unique properties. Members of the GABARAP family have been suggested to function late in autophagy, potentially promoting sealing of IMs or fusion of autophagosomes with lysosomes while LC3-proteins are believed to coordinate the expansion of autophagosomes, thus acting earlier than GABARAP proteins in the pathway (Weidberg *et al*., 2010). Importantly, overexpression or knockdown of one family member was shown to affect the expression levels of the other LC3 and GABARAP proteins (Weidberg *et al*., 2010). Therefore, our recent study (Jung *et al*., 2017) aimed to develop a screening platform for monitoring human ATG8 proteins (*i.e*., LC3B and GABARAP) at endogenous levels. This distinguishes our approach from other performed genome wide autophagy siRNA screens, in which overexpression of a GFP-tagged version of LC3B was employed (Orvedahl *et al*., 2011; McKnight *et al.*, 2012). Besides LC3B and GABARAP, we additionally included autophagy marker proteins for the initiation and maturation of autophagosomes such as WIPI2 (WD repeat domain phosphoinositide-interacting protein 2), ATG12 and STX17 (syntaxin 17), respectively. WIPI2 is recruited to phagophores by binding to the phospholipid phosphatidylinositol 3-phosphate (PI3P). Upon PI3P-binding, WIPI2 recruits the mammalian ATG8 lipidation complex comprised of the subunits ATG16L1, ATG5 and ATG12. Subsequently, LC3B is conjugated to PE and in turn can recruit several human ATG8-binding proteins including cargo receptors such as p62 (also known as SQSTM1), which lead to autophagy cargo engulfment. Fusion of autophagosomes with lysosomes requires the SNARE protein STX17. STX17 localizes to closed autophagosomes and associates with SNAP29 and VAMP8 on lysosomes (Ktistakis and Tooze, 2016). Finally, intraluminal components are lysosomally degraded. Application of several autophagic marker proteins such as ATG12, WIPI2 and STX17 in addition to LC3B and GABARAP potentially allows the elucidation of genes specific for early and late phases of the autophagic process. The identification of various known as well as enigmatic autophagy proteins verified our screening approach (Jung *et al*., 2017).

## Materials and Reagents

1. Pipette tips
2. 10 cm tissue culture dishes (Corning, Falcon^®^, catalog number: 353003)
3. 384-well imaging plates, CellCarrier-384 Black (PerkinElmer, catalog number: 6007550)
4. Cell culture microplate, 96-well, v-bottom (Greiner Bio One International, catalog number: 651180)
5. Disposable reagent reservoirs, 25 ml, sterile (VWR, catalog number: 613-1174)
6. Disposable reagent reservoirs, 100 ml, sterile (VWR, catalog number: 613-1172)
7. Aluminum sealing ape 96 100/CS (Corning, catalog number: 6570)
8. U2OS cells (ATCC, RRID:CVCL_0042)
9. Dulbecco’s modified Eagle’s medium (DMEM) (Thermo Fisher Scientific, Gibco™, catalog number: 41966029)
10. Fetal bovine serum (FBS) qualified, E.U.-approved, South America origin (Thermo Fisher Scientific)
11. Penicillin-streptomycin (P/S) (10,000 U/ml) (Thermo Fisher Scientific, Gibco™, catalog number: 15140122)
12. L-Glutamine 200 mM (Thermo Fisher Scientific, catalog number: 25030024)
13. 0.25% trypsin/EDTA (Thermo Fisher Scientific, Gibco™, catalog number: 25200056)
14. Dimethylsulfoxide (DMSO) (Sigma-Aldrich, catalog number: D8418)
15. siRNA library (GE Healthcare, Dharmacon, Cherry-picked library of siRNA pools for the proteins of interest)
16. Control siRNAs
  Non-targeting control siRNA (GE Healthcare Dharmacon, catalog number: D-001810-10-20) siRNAs for autophagy modulation:
  ATG12 (GE Healthcare Dharmacon, catalog number: J-010212-07)
  PIK3C3 (GE Healthcare Dharmacon catalog number: J-005250-09)
  RAB7A (GE Healthcare Dharmacon, catalog number: J-010388-07)
  Raptor siRNA sequence: GAUGAGGCUGAUCUUACAGUU (MWG)
17. Water DNase/RNase free, sterile (Thermo Fisher Scientific, Gibco™, catalog number: 10977035)
18. Sodium chloride (NaCl) (Sigma-Aldrich, catalog number: 31434)
19. Potassium chloride (KCl) (Sigma-Aldrich, catalog number: P9541-500G)
20. Sodium phosphate dibasic (Na_2_HPO_4_) (Sigma-Aldrich, catalog number: 71640-250G)
21. Potassium phosphate monobasic (KH_2_PO_4_) (Sigma-Aldrich, catalog number: P5379-100G)
22. Phosphate buffered saline (PBS) (Thermo Fisher Scientific, Gibco™, catalog number: 14190094)
23. Poly-L-lysine solution (Sigma-Aldrich, catalog number: P4707-50ML)
24. Optimal modified Eagle’s medium (Opti-MEM) (Thermo Fisher Scientific, Gibco™, catalog number: 31985062)
25. Lipofectamine RNAiMax (Thermo Fisher Scientific, Invitrogen™, catalog number: 13778150)
26. Paraformaldehyde (PFA) 4% in PBS (Santa Cruz Biotechnology, catalog number: sc-281692)
27. Triton X-100 (VWR, catalog number: 28817.295)
28. Bovine serum albumin (BSA) (Sigma-Aldrich, catalog number: A7906-100G)
29. Anti-ATG12 (Cell Signaling Technology, catalog number: 2010)
30. Anti-GABARAP (Abcam, catalog number: ab109364)
31. Anti-LC3B (MBL International, catalog number: PM036)
32. Anti-STX17 (Sigma-Aldrich, catalog number: HPA001204)
33. Anti-WIPI2 (Abcam, catalog number: ab105459)
34. Alexa Fluor 488 goat anti-rabbit IgG (Thermo Fisher Scientific, Invitrogen™, catalog number: A-11008)
35. Alexa Fluor 488 goat anti-mouse IgG (Thermo Fisher Scientific, Invitrogen™, catalog number: A-11001)
36. CellMask Deep red stain (Thermo Fisher Scientific, Invitrogen™, catalog number: H32721)
37. DRAQ5 (Cell Signaling Technology, catalog number: 4084S)
38. Phosphate-buffered saline (PBS, pH 7.4) (see Recipes)
  *Note: Plan ahead and order the total amount of reagents needed for the whole screen to avoid running out of any reagent while actually performing the screen*.

## Equipment

1. Pipette
2. Cell culture Incubator (37 °C, 5% CO_2_)
3. Centrifuge 5810R (Eppendorf, model: 5810 R, catalog number: 5811000010), rotor A-4-62 with well plate centrifugation inlays (Eppendorf, catalog number: 5810711002)
4. Cell culture sterile bench
5. Automated dispenser (MicroFill 96-/384-Well Microplate Dispenser) (BioTek Instruments, model: AF1000A)
6. 12-channel multichannel pipette (30-300 μl) (NeoLab, catalog number: E-1945)
  *Manufacturer: Eppendorf, model: Research^®^ plus*.
7. 12-channel multichannel pipette (10-100 μl) (NeoLab, catalog number: E-1943)
  *Manufacturer: Eppendorf, model: Research^®^ plus*.
8. Selma pipetting robot 96/25 μl (Analytik Jena, CyBio^®^, catalog number: OL7001-26-211) with 384-well plate adaptor (Analytik Jena, CyBio^®^, catalog number: OL7001-24-976)
9. TipTray 96; 25 μl, PCR certified, sterile (Analytik Jena, CyBio AG, catalog number: OL3800-25-733-P)
10. Neubauer counting chamber (Marienfeld-Superior, catalog number: 0640110)
11. Opera LX High Content Screening System with a 60x water-immersion objective and a robotic plate handler II 230 consisting of:
  - Opera LX 488/561/640 Microscope (PerkinElmer)
  - Water Objective 63x (PerkinElmer)
  - Plate handler II 230 (PerkinElmer)
    *Note: The Opera LX is discontinued*.

## Software

1. Excel (Microsoft)
2. Prism 4 (GraphPad)
3. Barcode generator (http://barcode.tec-it.com/de)
4. Acapella High Content Imaging Analysis Software (PerkinElmer)

## Procedure

*Note: All steps before cell fixation require processing under a sterile bench*.

**Figure 1:**
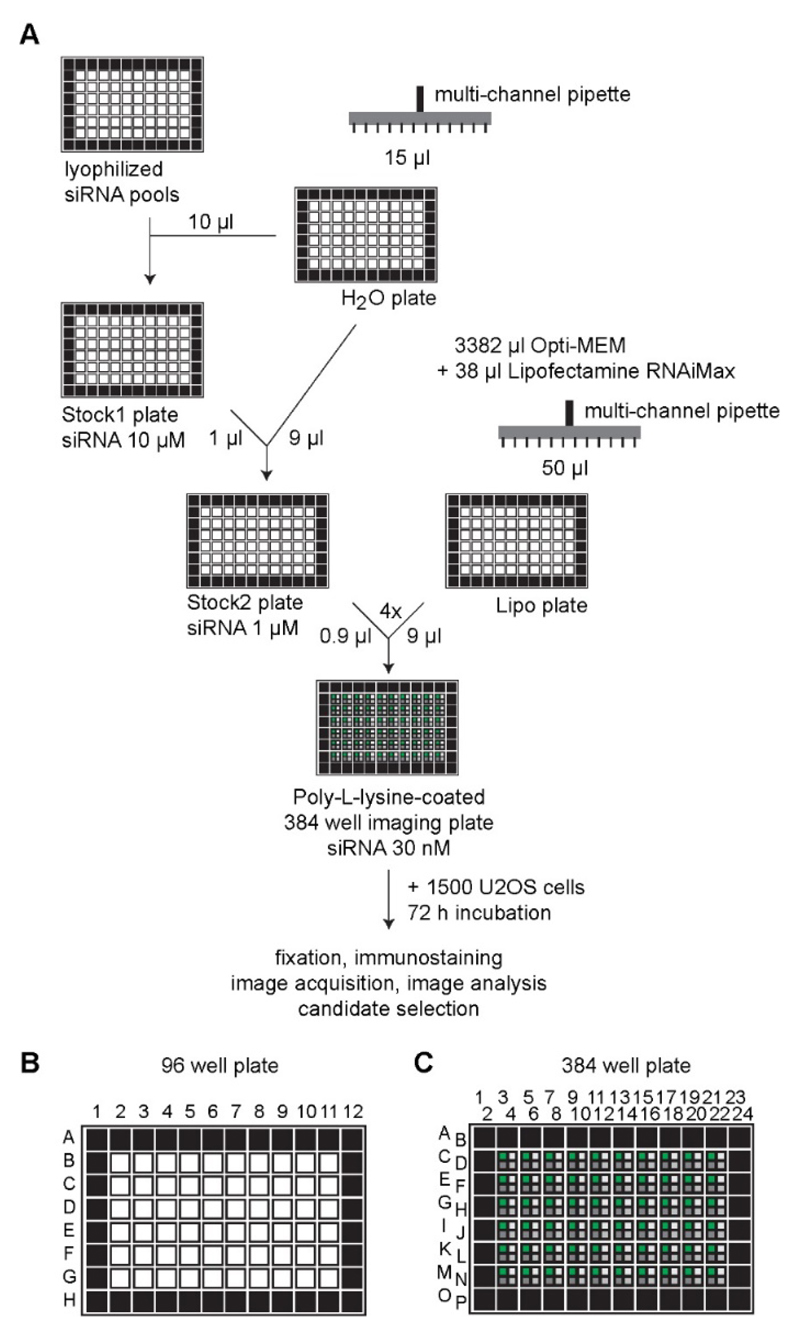
Scheme of the siRNA screening procedure. A. Lyophilized siRNAs in 96-well plates are solubilized and then diluted with water to receive a *Stock2 plate* containing siRNAs with a concentration of 1 μM, which is combined with a Lipofectamine RNAiMax-Opti-MEM-Mix (*Lipo plate*) and transferred to a 384-well imaging plate. U2OS cells are added prior to incubation of the 384-well plates. Finally, cells are fixed, immunostained and imaged followed by image and data analysis. B and C. It is recommended to avoid using the outer rim of the 96 (B) or 384 (C) well plates indicated by black wells to prevent edge effects.

- Maintaining U2OS cells
  1. Culture U2OS cells in DMEM, supplemented with 10% FBS, 2 mM glutamine as well as 1% P/S and incubated in a humidified cell culture incubator at 37 °C and 5% CO_2_.
  2. Frequently (~two times a week) passage cells after attaining approximately 80% confluency using 0.25% trypsin/EDTA.
  3. For long-term storage freeze cells at -150 °C in FBS containing 10% DMSO.
    Note: Freeze enough cell aliquots at the same time to perform the entire screen from one batch.
  4. Prepare enough 10 cm tissue culture dishes with U2OS cells to have sufficient cells of 70-80% confluency for the planned siRNA screen at the assay day. For two 384-well plates one 10 cm dish should be sufficient.
- Reverse siRNA transfection of U2OS cells (Figure 1A)
  1. Resuspend lyophilized siRNA pools (*Stock1 plate*)
    - Bring ordered 96-well plates containing the lyophilized siRNAs (0.1 nmol) from -80 °C storage to sterile bench after short centrifugation.
    - Equip pipetting robot (Selma) with new tips.
    - Dispense 15 μl RNase free water in every well of a 96-well v-bottom plate using a multi-channel pipette (*H_2_O plate*).
    - With the automated pipetting robot resuspend the lyophilized siRNA by transferring 10 μl RNase free water from the *H_2_O plate* into the siRNA containing 96-well plates from Dharmacon to obtain a 10 μM stock siRNA solution (*Stock1 plate*). Mix well by pipetting up and down using the automated pipetting robot in the ‘mixing cycles’ mode.
      *Notes*:
      - *Be careful to arrange the control siRNAs properly amongst the screening siRNAs (e.g., randomly across the plate but at least one control per lane) while ordering the 96-well plates with lyophilized siRNAs. Thereby, an extra step is avoided where the siRNAs have to be rearranged in a suitable sequence/order for the screen*.
      - *Do not use the outer rim of 96-well plates (A1-A12, H1-H12, B1, C1, …, B12, C12, …) to avoid edge effects (Figure 1B, black edges)*.
      - *Especially whilst using the automated pipetting robot constantly check the orientation of your plate. Always position the well A1 in one specific corner and remember the orientation. Most 96-well plates contain one notched corner for easier orientation*.
    - Discard *H_2_O plate* or also use for dilution of *Stock2 plate* when directly proceeding with protocol.
    - Cover *Stock1 plate* with an aluminum seal and store at -80 °C or proceed with protocol.
  2. Count U2OS cells with Neubauer counting chamber
    - Wash every 10 cm dish containing U2OS cells with 2 ml sterile PBS (see Recipes). Then, add 1.5 ml trypsin per dish and incubate for approximately 5 min at room temperature until the cells are detached.
    - Block trypsin activity by addition of 8 ml DMEM supplemented with 10% FBS, 2 mM glutamine but without P/S (DMEM(-)P/S).
    - Pool all cells for the screening assay in one flask (*e.g.*, common sterile 15 ml or 50 ml Falcons).
    - Transfer 10 μl of the pooled cell solution into a Neubauer counting chamber and count cells under a light microscope.
    - Dilute U2OS cells with DMEM(-)P/S to obtain a cell density of 7.14 x 10^4^ cells/ml (1,500 cells in 21 μl).
      *Note: Perform the cell counting before the 384-well imaging plate preparation to reassure the necessary number of cells is available*.
  3. Dilute siRNAs from *Stock1 plate* to obtain a 1 μM working solution in a *Stock2 plate.*
    - The amounts described here are sufficient for one full 384-well imaging plate loaded in quadruplicates. If more 384-well imaging plates are necessary for the screen, adjust the amounts accordingly.
    - Dispense 15 μl H_2_O in every well of a 96-well v-bottom plate using a multi-channel pipette (*H_2_O plate*).
    - With the automated pipetting robot using the ‘sample dilution’ mode pipette 9 μl RNase free water from the *H_2_O plate* and then add 1 μl from the *Stock1 plate* into the same tips and release both together in a new 96-well v-bottom plate to obtain a siRNA working solution of 1 μM (*Stock2 plate*). Mix well by pipetting up and down using the ‘mixing cycles’.
  4. Prepare reverse siRNA transfected U2OS cells in 384-well imaging plates with a final siRNA concentration of 30 nM.
    - Transfer 30 μl poly-L-lysine into every well of a 384-well imaging plate using a multi-channel pipette and incubate for at least 1 h at room temperature. Remove the poly-L-lysine from the 384-well imaging plate using a multi-channel pipette and discard. Let the 384-well imaging plate dry for a couple of minutes.
    - Mix 3,382 μl Opti-MEM with 38 μl Lipofectamine RNAiMax and place in reservoir (Lipo-Opti-Mix). For each 384-well this corresponds to 8.9 μl Opti-MEM and 0.1 μl Lipofectamine RNAiMax. The excess amount is prepared to assure enough liquid for the automated pipetting robot.
    - Transfer 50 μl Lipo-Opti-Mix in every well of a 96-well v-bottom plate using a multi-channel pipette (*Lipo plate*). Again, do not use the outer rim (Figures 1B and 1C).
    - Equip automated pipetting robot with new tips.
    - With the automated pipetting robot using the ‘sample dilution’ mode absorb 9 μl Lipo-Opti-Mix from the *Lipo plate* and subsequently 0.9 μl siRNA from the *Stock2 plate* into the same tips and release into the first replicate well of the 384-well imaging plate (Figure 1C, green wells).
      *Note: Again, check the orientation of your plate*.
    - Repeat 3 times to receive quadruplicates from one siRNA pool on the 384-well imaging plate (Figure 1C, grey wells).
    - Incubate siRNA-Lipo-Opti-Mix in 384-well imaging plate for 20 min at room temperature.
    - Dispense 21 μl DMEM(-)P/S including 1,500 U2OS cells per well (total volume 11.76 ml for one 384 plate) from a reservoir into each well of the 384-well imaging plate using a multi-channel pipette. Ensure that the liquid dropped to the well bottom or gently centrifuge plate if necessary. Gentle mixing by slowly pipetting up and down with a multi-channel pipette is possible but usually not necessary. While mixing, use new tips for every siRNA. 96- and 384-well plates can be centrifuged at 161 *x g* with the appropriate plate inlays for centrifuges.
      *Note: Given that cells quickly sink onto the reservoir bottom (only approximately 1 min equal distribution), mix cells in the reservoir regularly*.
    - Dispense 30 μl DMEM(-)P/S into each well of the 384-well edge (Figure 1C, black wells) using a multi-channel pipette to avoid edge effects.
  5. Incubate 384-well imaging plate in cell culture incubator for 72 h.
- Fixation and Immunostaining of cells
  1. Discard liquid from 384-well plates in proper cell culture waste.
  2. U2OS cells in the 384-well plates are fixed with 50 μl 4% PFA per well for 15 min at room temperature pipetted with a multi-channel pipette. Discard liquid in PFA waste in sealed glass bottles and check department instructions for proper waste disposal as suggested by the EH&S Chemical Waste Program.
  3. Wash cells for three times with 100 μl self-made PBS per well using a multi-channel pipette. Remove the PBS by turning the plate upside-down on top of a sink.
  4. Store plate containing 100 μl PBS per well at 4 °C or directly continue immunostaining protocol.
  5. Discard liquid.
  6. Using a multi-channel pipette, permeabilize cells with 50 μl 0.5% Triton X-100 in PBS per well and incubate for 10 min at room temperature. Discard liquid.
  7. Block cells with 100 μl 1% BSA in PBS per well transferred with a multi-channel pipette and incubate for 1 h at room temperature. Discard liquid.
  8. With an automated dispenser, wash cells once with 100 μl self-made PBS per well. Discard liquid.
  9. Prepare primary antibody solution (either anti-ATG12 1:50; anti-GABARAP 1:200; anti-LC3B 1:800; anti-STX17 1:250; or anti-WIPI2 1:500 in 0.1% BSA in PBS) and distribute 20 μl per well with a multi-channel pipette. Incubate cells for 1 h at room temperature and then discard liquid.
    *Note: Every 384-well plate is only stained with one primary antibody*.
  10. With an automated dispenser, wash cells for three times with PBS as described above.
  11. Prepare secondary antibody solution (anti-rabbit or anti-mouse Alexa Flour 488, 1:1,000 in 0.1% BSA in PBS) and distribute 20 μl per well with a multi-channel pipette. Incubate cells for 1 h at room temperature and then discard liquid.
  12. Prepare cytoplasmic staining solution (HSC CellMask Deep red stain 1:25,000,000 in 0.1% BSA in PBS) and distribute 20 μl per well with a multi-channel pipette. Incubate cells for 1 h at room temperature and then discard liquid.
  13. Prepare nuclear staining solution (DRAQ5, 1:5,000 in 0.1% BSA in PBS) and distribute 20 μl per well with a multi-channel pipette. Incubate cells for 10 min at room temperature and then discard liquid.
  14. With an automated dispenser, wash cells for three times with PBS as described above.
  15. After the third wash, distribute 100 μl sterile PBS per well with a multi-channel pipette and seal the 384-well imaging plate with an aluminum seal to avoid exposure to light. Store plate at 4 °C until image acquisition.
    Note: This last step is performed with a multi-channel pipette and sterile PBS to prolong storability of the imaging plates.

## Data analysis

**Figure 2:**
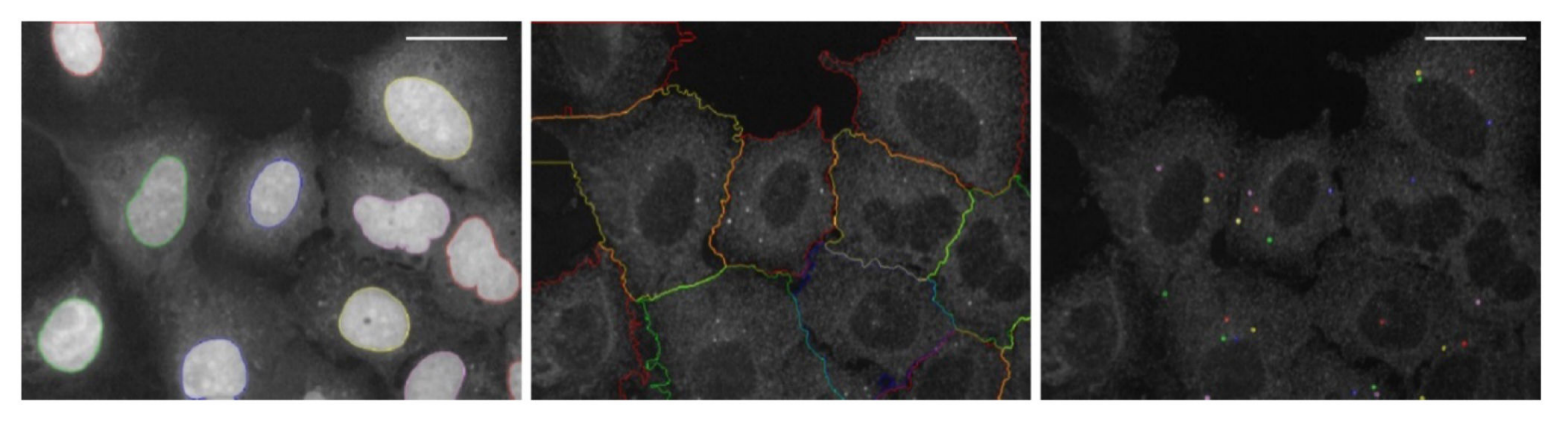
Image segmentation. Example images for proper image segmentation of the nuclei (left), the cytoplasm (middle) and the spots (right). Spots represent phagophores stained with anti-WIPI2 antibody. Scale bars = 40 μm.

- Image acquisition
  1. Choose the 60x water-immersion objective on a PerkinElmer’s Opera High Content Screening System microscope to receive a resolution suitable for intracellular spot detection.
    *Note: Phagophores and autophagosomes are detected as intracellular spots with the microscope*.
  2. Adjust the necessary laser intensity and plane height according to the used antibody.
    *Note: Acquire images sequentially, first in the 488-channel and then in the 633-channel*.
  3. Set the plate layout and select the number and distribution of fields per well.
    *Note: For U2OS cells 24 fields per well for 4 wells will approximately add up to more than 1,000 cells in all the images*.
  4. Save the settings.
  5. Set parameters for the Opera robotic plate handler (*e.g*., the location of the plates in the plate holder) and save settings to sequentially measure more than one plate.
  6. Label your plates with individual bar codes for each plate using http://barcode.tec-it.com/de. Print the bar codes and glue them onto the plates.
  7. Place all 384-well imaging plates into the plate holders.
    *Note: Be aware of the proper orientation of the plate*.
  8. Start bulk measurement with the saved settings to automatically image one plate after another with help of the robotic arm.
  9. Remove plates from stacker and keep at 4 °C or discard.
  10. Shut down the Opera microscope.
- Image analysis
  1. Open the Acapella High Content Imaging Analysis Software and load your image analysis script for spot detection.
  2. Set the general parameters, *e.g*., 488 equals channel one, where intracellular spots are detected, 633 equals channel two, where nuclei and cytoplasm are detected, according to the actual measurement.
  3. Set script parameters including contrast, area and the detection algorithm to properly segment nuclei, cytoplasm and spots in every cell. See Figure 2 for example images.
  4. Save all settings and load these parameters into the script next time.
  5. Run the analysis for a couple of images (well mode), which are obtained from assay specific control siRNAs and check the output results. Perform minor adjustments to the parameters if necessary (*e.g*., contrast can slightly vary for plates immunostained on different days).
    *Note: Especially in the script preparation phase always compare the output results with a manual count of your acquired images. Manual counting means that a person actually counts the number of spots by hand*. *If the image quantification output results don’t match your own manual count, adjust the parameters until you are satisfied with the output result.*
  6. Run the script in batch mode for the all the images in the whole 384-well imaging plate and save the output results as Excel file.
  7. Representative example images for all the different antibody stainings are shown in Jung *et al*., 2017.
- Candidate determination
  1. Average raw data of quadruplicates for every siRNA in Excel (in this assay the number of spots) and calculate the standard deviation.
  2. Normalize the output results (number of spots) per siRNA to non-targeting control siRNA for every 384-well plate. See Table 1 for an imaginary data example.
  3. Compare the fold-change of all tested siRNAs from all plates with each other.
  4. Classify candidate siRNAs with your selected method *e.g*., the standard deviation criteria. Here, siRNAs, whose fold-change differ for two or three standard deviations from the normalized sicon (WIPI2 and ATG12 = 3; LC3B, GABARAP and STX17 = 2) are selected as candidates. See Table 1 for an imaginary data example.
  5. Excel can also be used to order and rank the candidates according to the fold-change difference to elucidate the top candidates.
  6. Prism 4 (GraphPad) was applied to generate diagrams and for statistical analysis (ANOVA).
  7. Representative example graphs are shown in Jung *et al*., 2017.
- Perform a deconvolution screen with four individual siRNAs per gene for your top candidates using the same procedure as for the siRNA pool screen described above.
  1. Classify validated candidates, which differ in the standard deviation criterion as above for three out of four siRNAs per gene.
  2. Exclude toxic siRNAs, which showed obvious changes in number of cells as well as in the intensity and area of the nucleus or of the cytoplasm. Remove genes with more than one cytotoxic siRNA for further analysis.
- General considerations for screening approaches
  1. Think about the proper cell line for the screening approach. The human osteosarcoma cell line used for this image-based autophagy screen is adherent and provides a big cytoplasm, which makes this cell line appropriate for immunostaining and imaging. Furthermore, autophagy can be induced as well as inhibited in U2OS cells. In addition, siRNA transfection is very efficient in this cell line. According to these properties, U2OS cells are well suited for an image-based autophagy siRNA screen. We assume that *e.g*., HeLa, A549 or LN229 cells would also be suitable for this screening approach.
  2. Using your method of choice including immunofluorescence, immunoblot or RT-qPCR, check that your assay control siRNAs actually provide an efficient knockdown of the intended target genes and induce the expected phenotype in your chosen cell line.
  3. Elucidate the number of cells per well necessary for a good but not crowded well coverage with your chosen cell line (*e.g*., ~80% well coverage). As explanation, a lot of empty space in between cells might cause long image acquisition times and image analysis artefacts. Usage of too many cells might cause them to grow on top of each other, which again might introduce image analysis artefacts. To determine the proper cell number, transfect cells with control siRNAs and use several different cell densities (*e.g*., 500-10,000 cells per well). Perform staining, imaging and analysis to determine the proper number of cells for seeding.
  4. Elucidate the suitable transfection reagent and siRNA (different companies) for your application. Therefore, transfect U2OS cells with different transfection reagents and assay control siRNAs. Perform staining, imaging and analysis to determine the best transfection reagent, which does not interfere with the selected screening pathway (autophagy in this case). Also, different amounts of the selected transfection reagent can be used for optimal knockdown efficiencies.
  5. In this image-based autophagy screen immunofluorescence staining of proteins at endogenous levels with antibodies is applied. To reduce costs the highest possible antibody dilution with a detectable immunofluorescence signal should be determined. Therefore, seed U2OS cells in your imaging plate and stain using a dilution series of your antibody. Perform imaging and analysis to determine the lowest amount of antibody necessary. The dilution series can also be performed with secondary antibodies, the nuclear and the cytoplasm stain.
  6. While determining the proper ‘wet lab’ screening condition, start the script development. Elucidate the correct parameters to detect your phenotype, in this case number of spots. Always compare the number of spots calculated by the script with the actual pictures for a few randomly selected images to make sure that the script is counting properly.
  7. Apply normalization techniques thoroughly (for example, normalization per plate to non-targeting control siRNAs) and carefully select the criteria to define candidates. The 2-3 times fold-change of the aberration of standard deviations is often applied. Other potential methods are Z- and B-score normalization, especially for genome-wide siRNA screens.
  8. Imply cytotoxicity measures into the script, since toxic siRNAs can result in false positive hits. For example, compare number of cells and other general criteria such as area and intensity of the nucleus and the cytoplasm and remove siRNAs with an obvious aberration.
  9. Apply statistics to control your screening conditions and off-target effects such as the correlation of output results between repetitions or between individual and pool siRNAs, respectively.
  10. Think about and establish downstream assays to mechanistically validate your candidates. Typical methods to study autophagy modulation include GFP/RFP-based autophagy flux assays and immunoblot analysis with autophagy markers such as LC3B or p62. Further on, localization of the candidate protein as well as co-localization of the candidate with early and late autophagic markers can be determined by confocal microscopy.

## Recipes

1. Phosphate-buffered saline (PBS, pH 7.4)
  137 mM NaCl
  2.7 mM KCl
  9 mM Na_2_HPO_4_
  3 mM KH_2_PO_4_

## Figures and Tables

**Table 1:** Imaginary data example for the staining with LC3B based on Jung *et al*. (2017), to explain data normalization and candidate selection Number of spots were quantified using the Acapella Software. For normalization the number of spots of every siRNA was divided by the number of spots of the non-targeting control siRNA. This yielded a fold change of 3.2 or 0.3 for the positive or negative control siRNAs, respectively. Furthermore, the target siRNAs 2 and 3 would have been selected as candidates for LC3B, according to the standard deviation criterion. As example the average standard deviation for LC3B was approximately 23%, a selected increasing or decreasing candidate siRNA comprises a fold-change of 1.46 or higher (1.0 + 2 x 23%) or 0.54 or lower (1.0 - 2 x 23%), respectively.

LC3B	Non-targeting siRNA control	Positive control siRNA RAB7	Negative control siRNA ATG12	Target siRNA 1	Target siRNA 2
**Spot numbers**	6.44	20.61	1.93	9.40	3.48
**Normalized spot numbers**	1.00	3.20	0.30	1.46	0.54

## References

[1] Jung J, Nayak A, Schaeffer V, Starzetz T, Kirsch AK, Muller S, Dikic I, Mittelbronn M, Behrends C (2017). Multiplex image-based autophagy RNAi screening identifies SMCR8 as ULK1 kinase activity and gene expression regulator. Elife.

[2] Ktistakis NT, Tooze SA (2016). Digesting the expanding mechanisms of autophagy. Trends Cell Biol.

[3] McKnight NC, Jefferies HB, Alemu EA, Saunders RE, Howell M, Johansen T, Tooze SA (2012). Genome-wide siRNA screen reveals amino acid starvation-induced autophagy requires SCOC and WAC. EMBO J.

[4] Orvedahl A, Sumpter R, Xiao G, Ng A, Zou Z, Tang Y, Narimatsu M, Gilpin C, Sun Q, Roth M, Forst CV (2011). Image-based genome-wide siRNA screen identifies selective autophagy factors. Nature.

[5] Weidberg H, Shvets E, Shpilka T, Shimron F, Shinder V, Elazar Z (2010). LC3 and GATE-16/GABARAP subfamilies are both essential yet act differently in autophagosome biogenesis. EMBO J.

